# Collaborative Admission Pathway for Service Users with Complex Emotional Needs

**DOI:** 10.1192/bjo.2026.11372

**Published:** 2026-06-30

**Authors:** Omar Dwekat, Teresa Luu, Kubranur Durmaz, David Mirfin

**Affiliations:** Surrey and Borders Partnership NHS Trust, Surrey, United Kingdom

## Abstract

**Aims:**

This project aimed to develop and implement a structured inpatient admission support leaflet for individuals with Emotionally Unstable Personality Disorder (EUPD) and complex emotional needs (CEN), in line with NICE guidance (Borderline Personality Disorder: Recognition and Management, recommendation 1.4.1.3).

The leaflet outlines expectations, support processes, and a seven-day admission framework, integrating collaborative care planning, communication strategies, sensory needs, and a colour-coded Mood Intensity Meter.

The objectives were to improve clarity of inpatient processes, enhance engagement and emotional communication, promote collaborative risk and discharge planning, and support safe, time-limited admissions within a trauma-informed, person-centred model of care.

**Methods:**

We piloted this intervention on Polaris Ward at Silverwood Hospital. Within the inpatient population we identified those with a diagnosis of EUPD with CEN and measured the average number of days from admission to discharge; patient wellbeing as reported through nursing staff reports and weekly ward reviews; and to what extent patients worked collaboratively with the clinical team. This data was collected between August 2025 to end January 2026. The CEN brochure was written based on NICE guidelines and trust policies and was presented at the community meeting to obtain feedback from service users with EUPD. This was distributed amongst new admissions fitting the patient population from February 2026 and the same parameters re-audited from February 2026 to August 2026.

**Results:**

Introduction of the CEN brochure and structured collaborative discussions was associated with improvements in several key areas. Nursing reports and ward reviews highlighted notable increases in patient engagement, with more patients actively participating in goal setting and ward round discussions. Patients demonstrated improved emotional regulation and wellbeing, reflected in more stable ward presentations and fewer episodes requiring unplanned staff intervention. Collaborative care planning improved as patients more consistently understood the purpose and expected duration of their admission, facilitating clearer shared expectations. While length of stay showed positive reductions, the greater impact appeared in the quality of collaboration and patient experience, suggesting that the intervention enhanced both the therapeutic relationship and the efficiency of care planning.

**Conclusion:**

This intervention suggests that providing structured, accessible information tailored to patients with EUPD and CEN early in their admission can improve collaborativeworking, patient understanding and reduce the length of admission. This intervention was piloted in one inpatient working age ward, and should be trialled in further wards.

